# Bilateral juvenile osteochondrosis dissecans in monozygotic twins: a case report

**DOI:** 10.1186/s13018-024-04683-2

**Published:** 2024-04-01

**Authors:** Luca Bausch, Monika Probst, Lorenz Fritsch, Julian Mehl, Sebastian Siebenlist, Lukas Willinger

**Affiliations:** 1 Department of Sports Orthopaedics, Technical University Munich, Ismaninger Str. 22, 81675 Munich, Germany; 2 Department of Diagnostic and Interventional Neuroradiology, Klinikum rechts der Isar, Technical University Munich, Munich, Germany

**Keywords:** Osteochondrosis dissecans, Osteochondral lesion, Femoral condyle, AOCD, JOCD

## Abstract

**Introduction:**

The etiology of osteochondrosis dissecans (OCD), a chondropathy associated with detachment of the subchondral bone and the overlaying cartilage, is not yet fully understood. While repetitive physical exercise-related stress is usually assumed to be the main risk factor for the occurrence of OCD, genetic predisposition could have an underestimated influence on the development of the disease.

**Case report:**

We report a case of monozygotic twins with almost identical stages of bilateral osteochondrosis dissecans of the knee joint. In both patients, initially, a unilateral lesion occurred; despite restricted physical exercise, in the further course of the disease a lesion also developed on the contralateral side. While the lesion found most recently demonstrated an ongoing healing process at a 6-month follow-up, the other three lesions showed a natural course of healing under conservative treatment with significant clinical as well as radiological improvements after one year and complete consolidation in magnetic resonance imaging (MRI) after 2 years.

**Conclusion:**

There could be a genetic component to the development of OCD, although this has not yet been proven. Based on a two-year MRI follow-up, we were able to show the self-limiting characteristics of juvenile osteochondrosis dissecans.

## Introduction

Osteochondrosis Dissecans (OCD) is a multifactorial chondropathy associated with the risk of detachment of a loose body [2]. The disease occurs mainly in the knee, elbow or ankle joints and can be classified as a juvenile form (before closure of the epiphyseal joints, JOCD) and an adult form (after closure of the epiphyseal joints, AOCD) [4]. The knee joint is the most commonly affected joint [8]: Here, in the age group of the highest incidence from 12 to 19 years, 63.3% of the lesions are seen in the medial femoral condyle and 32.5% in the lateral femoral condyle. 7.3% of patients have bilateral lesions [11]. In addition, males have a fourfold increased risk of developing OCD compared to females [4].

Besides biomechanical factors (discoid meniscus configuration, varus malalignment [3], impingement of the intercondylar eminence), acute trauma, exercise-related stress and biological predisposition (deficient enchondral ossification or endocrine factors such as vitamin D deficiency), there are genetic risk factors [1], which are the current subject of research.

The etiology of the disease has not yet been sufficiently established scientifically, yet the so-called microtrauma theory is widely accepted [17]. According to this theory, repetitive physical exercise-related stress and the resulting stress response cause microtrauma in the bone bed, which can ultimately lead to impaired blood flow with subsequent risk of detachment of a cartilage fragment [12].

Although the stability of such a detachment can be examined most reliably using arthroscopy [10], magnetic resonance imaging is the diagnostic gold standard due to its very high sensitivity and specificity in the detection of OCD [8].

Osteochondral lesions (OCL) are classified on MRI according to Dipaola [5] as follows:

The prognosis of the juvenile form of OCD is usually better than in adults, as it is self-limiting in most cases and heals with closure of the growth plate [16]. While the non-operative therapeutic approach based on refraining from physical exercise and rest often leads to significant improvement of JOCD within one year, additional surgical procedures usually have to be considered for AOCD. Depending on the size and stability of the lesion as well as the epiphyseal joint status, various procedures such as retrograde drilling, refixation of the loosened fragment or even regenerative cartilage therapy are available [4].

The aim of the study was to investigate the clinical and radiological course of bilateral juvenile OCD in monozygotic twins. In addition, by means of regular MRIs, the study aimed to show the self-limiting process as well as the morphological imaging changes of the disease over time.

## Case history

### Twin 1

The 10-year-old pupil presented with exercise-induced pain on the inner side of the right knee joint, especially after having played football, which also occurred in an identical manner on the left side at intervals of a few months. There was no pain at rest, the clinical examination of both knee joints was normal, and the patient did not recall any trauma in either case.

### Twin 2

The 13-year-old patient presented with physical exercise-induced complaints of the left knee joint equivalent to those of his brother, which also developed on the contralateral side six months later. Both knee joints showed normal range of motion and no instability in the clinical examination and there was no trauma-related origin in either case. Here, too, a contralateral lesion developed even though, due to the symptoms on the left knee, the patient adhered to a strict no-sports regimen and thus lesion-promoting stress was avoided.

### Imaging

#### Twin 1

**Fig. 1:**
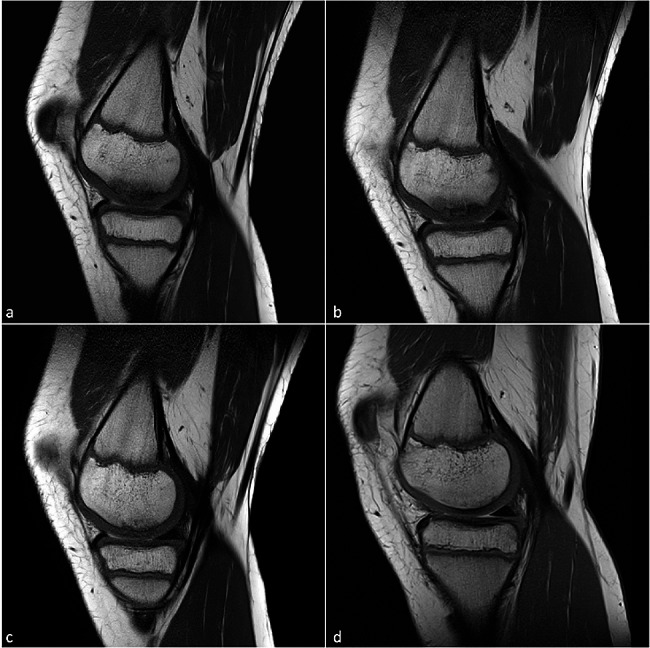
T1-weighted turbo spin echo sequences (T1-TSE) follow-up MR images of osteochondrosis dissecans of the right knee joint of twin 1 in the sagittal plane: from the stage at the onset of symptoms *a)*, there is a decrease in lesion size and perifocal edema after *b)* six months and *c)* fourteen months until complete consolidation of the lesion under conservative treatment after *d)* two years of follow-up. There is a decrease in reactive cartilage thickening with a continuous cartilage surface

Right knee joint: The MRI scan showed an osteochondral lesion on the medial femoral condyle. The overlying cartilage showed reactive thickening in accordance with stage II OCD and there was perifocal edema. Four follow-up MRI scans were conducted over a period of two years. Over time, there was a decrease in the size of the lesion, a decrease in the perifocal edema and a consistently stable chondral bone cover, which showed no signs of detachment of an osteochondral fragment (Fig. 1).

**Fig. 2:**
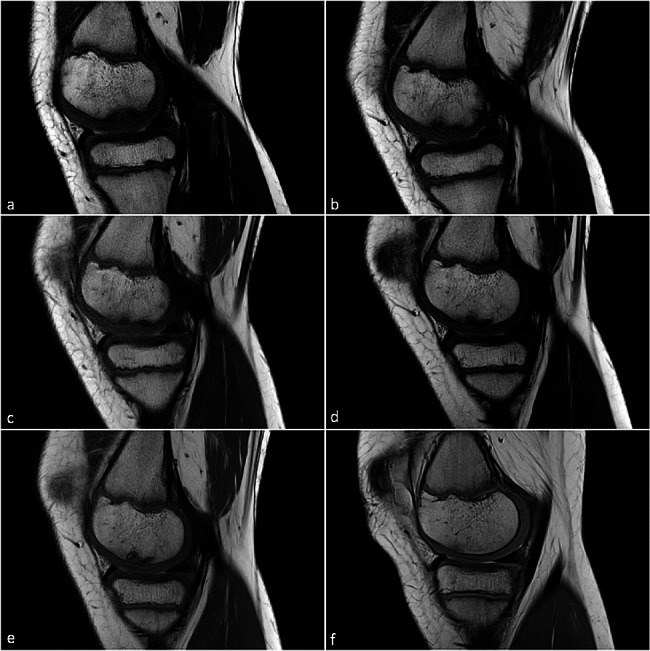
T1-weighted turbo spin echo sequences (T1-TSE) follow-up MR images in the sagittal plane of osteochondrosis dissecans of the left knee joint of twin 1 from *(a)* symptom onset, after *(b)* three months, *(c)* six months, *(d)* one year, *(e)* one and a half years to complete regression of the lesion after a little more than *(f)* two years. The cartilage surface remains intact at all times, the reactive cartilage thickening as well as the perifocal edema regress completely

Left knee joint: Here, six follow-up MRI scans were conducted over a period of two years. The diagnosis established was osteochondrosis dissecans in loco typico (medial femoral condyle) characterized by perifocal edema and a slightly thickened cartilage cover in a stable, exclusively subchondral lesion (Fig. 2).

### Twin 2

**Fig. 3:**
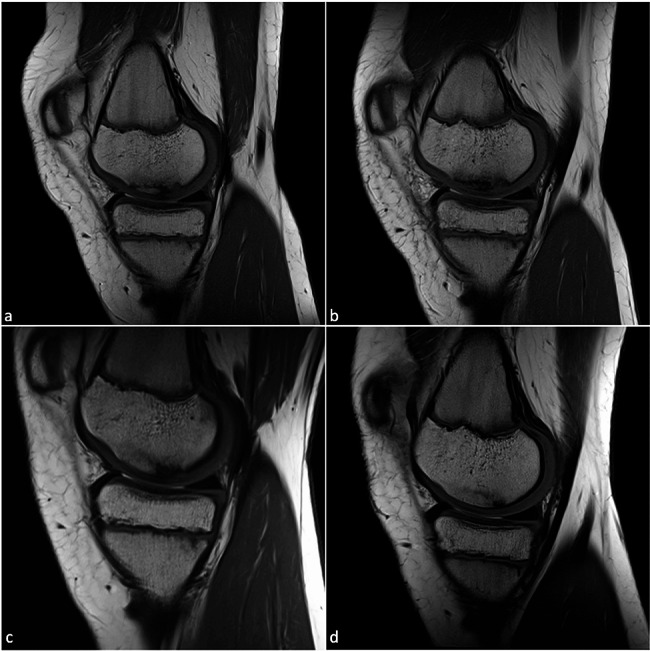
T1-weighted turbo spin echo sequences (T1-TSE) follow-up MR images in the sagittal plane of osteochondrosis dissecans of the left knee joint of twin 2 after *(a)* first clinical presentation, *(b)* three months, *(c)* seven months and *(d)* 12 months. The cartilage cover is intact at all times, the defect size is significantly reduced over time and the bone structure appears almost completely regenerated in the last MRI

Left knee joint: There was a 12 × 25 mm OCL at the medial femoral condyle with minimal adjacent marrow edema and subchondral demarcation with a continuous bone cover. In addition, Osgood-Schlatter disease was diagnosed. Four follow-up images were taken in a period of twelve months after the initial diagnosis and showed a defect in consolidation, which was still covered entirely by cartilage and showed no signs of detachment. The lesion was almost completely consolidated in the last picture (Fig. 3).

**Fig. 4:**
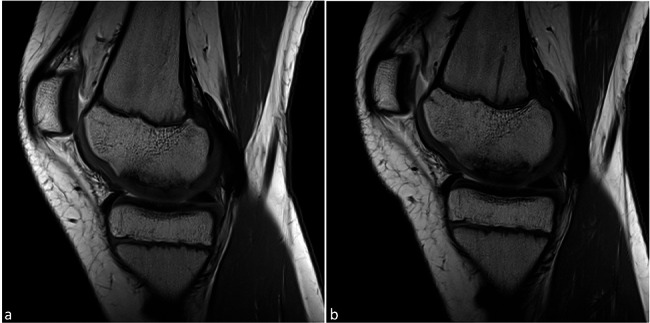
Typical presentation of T1-weighted turbo spin echo sequences (T1-TSE) follow-up MR images in the sagittal plane of stage I osteochondrosis dissecans in the right knee joint of twin 2: a stable lesion with moderate osteolysis and reactively thickened but intact cartilage cover from *(a)* the stage at the onset of symptoms and *(b)* after 5 months

Right knee joint: The MRI scan of February 2023 showed a stable OCL of 16 × 30 mm with surrounding marrow edema and continuous cartilage cover (Fig. 4). The MRI performed five months later displayed a slight decrease in edema with the defect size and morphology of the lesion remaining almost unchanged.

### Treatment and course of disease

Both patients were recommended to undergo non-operative treatment by refraining from physical exercise for 6 months after detection of a lesion in the respective knee. Especially playing football and school sports including jumps and axial loads should have been avoided, but no restrictions were given for everyday activities with full weight-bearing allowed. In order to promote bone metabolism, an additional substitution of vitamin D and calcium was recommended for twin 2. The healing process in twin 1 was satisfactory both radiologically and clinically: After one year, consolidation of the lesion on the right knee had occurred to the extent that sports activities could have been resumed. However, due to the contralateral lesion, returning to sports had to be postponed for another 6 months until OCD consolidation was confirmed by MRI. In twin 2, the left knee joint showed a clinically and radiologically satisfactory healing progress after 12 months. Here, an analogous healing process of the right knee is to be expected, but after 5 months, evident morphological imaging improvements of the lesion are yet to be seen.

## Discussion

The main findings of this case report were that the bilateral OCD lesions of two monozygotic twins displayed a very similar and satisfactory clinical and radiological healing process with conservative treatment. The lesions’ morphology was comparable in terms of size, location at the medial condyle of the femoral bone and the healing course. Plus, the clinical course in both patients as well as their symptoms were also almost identical. It is to be noted, that despite conservative treatment and sports restriction, an osteochondral lesion also appeared on the opposite side suggesting a genetic preposition for the development of an OCD lesion in these patients 1.

**Table 1:** Staging system for characterizing osteochondral lesions

Stage	Description
1	Thickening of the cartilage, cartilage surface intact
2	Breached articular cartilage with low-signal defect margin around the fragment as an indication of fibrous attachment
3	Breached articular cartilage with high-signal fluid margin around the fragment
4	Detached osteochondral fragment with destruction of the articular surface

At present, there are no measures to prevent the disease, which usually occurs in adolescence, and the etiology of OCD is not yet sufficiently understood. The significance of the clinical picture becomes clear by the high incidence of 11–29 cases per 100,000 in children and adolescents [11, 14].

A genetic predisposition seems to have a possible influence on the development of OCD, but it has not yet been proven to be the sole reason for the development of the disease.

Based on the current state of research on the correlation between genetics and OCD, it becomes evident that there are many indications that, taken together, suggest a genetic etiology. In the literature, detached OD fragments have already been examined [18], genome-wide sequencing has been conducted in humans and animals [20], familial OCD has been demonstrated and studied in families across generations [7], the frequent occurrence of OCD in genetic, syndromal disorders have been shown [7] and several cases of OCD in twins, as in our case, have been described [16, 17].

Genome-wide association studies (GWAS) have already identified the aggrecan gene as a potential risk gene causing OCD, since missense mutations frequently occur in affected families and lead to a loss of function of the aggrecan protein, which is essential for the cartilage matrix, and thus leads to instability of the cartilage surface [19]. GWAS appear to be delivering the greatest advancements in genetic research on OCD, especially as these studies can now be conducted more frequently due to decreasing costs [20]. Potential genes of interest in animals have already been identified, which are intended to be examined in human tissue samples in the future [20]. In addition to that, Skagen et al. analyzed detached OCD fragments, discovering that the development of OCD is caused by a change in the synthesis of chondrocyte matrix [18], in which aggrecan plays an important role. Furthermore, the genome-wide methylation status seems to play a crucial role in gene expression in affected patients, which has already been described as a triggering factor for bone demineralization through altered gene expression in research on the causes of osteoporosis [2].

It seems interesting that one of the twins discussed in the case report also showed an Osgood-Schlatter disease in addition to OCD. This disease, which also occurs in adolescence, is directly associated with physical exercise and overloading of the immature cartilage-bone junction. However, the theory that OCD develops mainly due to sports overload is contradicted by the fact that both twins developed an osteochondral lesion on the contralateral side in spite of the recommended sports restriction due to the initially unilateral lesion. Therefore, a multifactorial genesis of the disease is currently assumed [3], which, in the case presented, manifests itself through the risk factors of male gender, physical exercise, young age and genetic predisposition.

Etiology is not yet sufficiently understood which could be due to the fact that OCD is usually only recognized at a late stage. On the one hand, this is related to the varying clinical presentation of the disease, on the other hand, to the fact that patients often do not remember a trauma-related genesis [4] and no screening method has been established so far to filter out risk patients at an early stage.

If the diagnosis is already established in adolescence, a good healing potential with a mostly self-limiting course can be expected [16]. Refraining from physical exercise and resting the affected limb can be considered to be the most important pillars of conservative treatment, as the symptoms seem to worsen under the influence of axial load and physical exercise [8]. With the goal of symptom freedom and radiologically proven consolidation of the lesion, conservative treatment should be continued for at least three to six months and longer until the goal is achieved [4].

AOCD usually develops based on a latent, unrecognized JOCD [4]. The appearance of OCD on the medial femoral condyle, as in our case, is consistent with the current literature describing the knee joint as the most affected joint of the human body [8]. In addition to the knee joint, however, the talus and elbow joint are primarily affected by JOCD [4], whereas the hip and shoulder joints are rarely affected [8]. According to Chau et al., JOCD develops in 9.5 to 29 of 100,000 knees, 2.2 of 100,000 elbows, and 2 to 4.6 of 100,000 ankles. Boys seem to have a higher risk of developing JOCD in the knee (4 times) and elbow (7 times) than girls, whereas girls have a higher risk of developing OCD in the talus (1.5 times) [4].

If the initial diagnosis is established after the epiphyseal joints have closed, the prognosis is usually worse, as an unstable or detached osteochondral fragment is often already present [9]. In adults, surgical treatment is often recommended for specific lesions to alleviate symptoms, avoid joint destruction and prevent the possible late effects of arthrosis [4]. The exact procedure is planned depending on the size of the lesion, the stability of the lesion and the symptoms after conservative treatment of at least six months. Even though surgical treatment by means of arthroscopy is considered to be joint-sparing, it still represents an invasive intervention that should be avoided through targeted early detection measures and strict conservative treatment. Another preventive and therapeutic approach could be the substitution of vitamin D: in 75.4–97.5% of those affected by OCD, a lowered serum vitamin D level (< 30ng/mL) and in 35.4–60% of patients a significant vitamin D deficiency (< 20ng/mL) was reported [6, 15]. Such hypovitaminosis can be confirmed by conventional laboratory analysis and thus represents a risk factor that could be detected comparatively easily and early and remedied by supplementing the vitamin. In addition, vitamin D substitution could prevent the progression of OCD and promote self-limitation of the disease [13]. However, further studies that may examine the effect of physical training or of other substances influencing bone metabolism in patients with OCD as well as studies providing evidence of a genetic etiology need to be conducted.

## Conclusion

In conclusion, due to the similar intervals of occurrence and of the course of the disease in both twins, there could be a genetic component to the development of OCD, although this has not yet been proven. In both cases, conservative therapy did not prevent the occurrence of the lesion on the contralateral side. However, MRI showed gradual and satisfactory healing process over time with complete consolidation of the OCD lesions.

Figure 1 T1-weighted turbo spin echo sequences (T1-TSE) follow-up MR images of osteochondrosis dissecans of the right knee joint of twin 1 in the sagittal plane: from the stage at the onset of symptoms *a)*, there is a decrease in lesion size and perifocal edema after *b)* six months and *c)* fourteen months until complete consolidation of the lesion under conservative treatment after *d)* two years of follow-up. There is a decrease in reactive cartilage thickening with a continuous cartilage surface.

## Data Availability

All the data are contained within this manuscript.

## References

[1] Andriolo L, Candrian C, Papio T, Cavicchioli A, Perdisa F, Filardo G. Osteochondritis dissecans of the knee - conservative treatment strategies: a systematic review. Cartilage. 2019;10(3):267–77. 29468901 10.1177/1947603518758435 PMC6585290

[2] Bates JT, Jacobs JC Jr., Shea KG, Oxford JT. Emerging genetic basis of osteochondritis dissecans. Clin Sports Med. 2014;33(2):199–220. 24698039 10.1016/j.csm.2013.11.004 PMC3976886

[3] Bruns J, Werner M, Habermann C. Osteochondritis dissecans: etiology, Pathology, and imaging with a special focus on the knee Joint. Cartilage. 2018;9(4):346–62. 28639852 10.1177/1947603517715736 PMC6139592

[4] Chau MM, Klimstra MA, Wise KL, Ellermann JM, Tóth F, Carlson CS, et al. Osteochondritis dissecans: current understanding of Epidemiology, etiology, management, and outcomes. J Bone Joint Surg Am. 2021;103(12):1132–51. 34109940 10.2106/JBJS.20.01399 PMC8272630

[5] Dipaola JD, Nelson DW, Colville MR. Characterizing osteochondral lesions by magnetic resonance imaging. Arthroscopy. 1991;7(1):101–4. 2009106 10.1016/0749-8063(91)90087-E

[6] Fraissler L, Boelch SP, Schäfer T, Walcher M, Arnholdt J, Maier G, et al. Vitamin D Deficiency in patients with idiopathic and traumatic Osteochondritis dissecans of the Talus. Foot Ankle Int. 2019;40(11):1309–18. 31370694 10.1177/1071100719864325

[7] Gornitzky AL, Mistovich RJ, Atuahuene B, Storey EP, Ganley TJ. Osteochondritis dissecans lesions in Family members: does a positive family history impact phenotypic potency? Clin Orthop Relat Res. 2017;475(6):1573–80. 27600715 10.1007/s11999-016-5059-x PMC5406325

[8] Heyworth BE, Kocher MS. Osteochondritis dissecans of the knee. JBJS Rev. 2015;3(7). 10.2106/JBJS.RVW.N.00095 27490144

[9] Hughston JC, Hergenroeder PT, Courtenay BG. Osteochondritis dissecans of the femoral condyles. J Bone Joint Surg Am. 1984;66(9):1340–8. 6501330 10.2106/00004623-198466090-00003

[10] Jacobs JC Jr., Archibald-Seiffer N, Grimm NL, Carey JL, Shea KG. A review of arthroscopic classification systems for osteochondritis dissecans of the knee. Orthop Clin North Am. 2015;46(1):133–9. 25435042 10.1016/j.ocl.2014.09.009

[11] Kessler JI, Nikizad H, Shea KG, Jacobs JC Jr., Bebchuk JD, Weiss JM. The demographics and epidemiology of osteochondritis dissecans of the knee in children and adolescents. Am J Sports Med. 2014;42(2):320–6. 24272456 10.1177/0363546513510390

[12] Kocher MS, Tucker R, Ganley TJ, Flynn JM. Management of osteochondritis dissecans of the knee: current concepts review. Am J Sports Med. 2006;34(7):1181–91. 16794036 10.1177/0363546506290127

[13] Krause M, Lehmann D, Amling M, Rolvien T, Frosch KH, Püschel K, et al. Intact bone vitality and increased accumulation of nonmineralized bone matrix in biopsy specimens of juvenile osteochondritis dissecans: a histological analysis. Am J Sports Med. 2015;43(6):1337–47. 25759459 10.1177/0363546515572579

[14] Lindén B. The incidence of osteochondritis dissecans in the condyles of the femur. Acta Orthop Scand. 1976;47(6):664–7. 1015263 10.3109/17453677608988756

[15] Maier GS, Lazovic D, Maus U, Roth KE, Horas K, Seeger JB. Vitamin D Deficiency: the missing etiological factor in the development of Juvenile Osteochondrosis dissecans? J Pediatr Orthop. 2019;39(1):51–4. 28009798 10.1097/BPO.0000000000000921

[16] Mei-Dan O, Mann G, Steinbacher G, Cugat RB, Alvarez PD. Bilateral osteochondritis dissecans of the knees in monozygotic twins: the genetic factor and review of the etiology. Am J Orthop (Belle Mead NJ). 2009;38(9):E152–5. 19911106

[17] Onoda S, Sugita T, Aizawa T, Ohnuma M, Takahashi A. Osteochondritis dissecans of the knee in identical twins: a report of two cases. J Orthop Surg (Hong Kong). 2012;20(1):108–10. 22535824 10.1177/230949901202000123

[18] Skagen PS, Horn T, Kruse HA, Staergaard B, Rapport MM, Nicolaisen T. Osteochondritis dissecans (OCD), an endoplasmic reticulum storage disease? A morphological and molecular study of OCD fragments. Scand J Med Sci Sports. 2011;21(6):e17–33. 20561273 10.1111/j.1600-0838.2010.01128.x

[19] Stattin EL, Wiklund F, Lindblom K, Onnerfjord P, Jonsson BA, Tegner Y, et al. A missense mutation in the aggrecan C-type lectin domain disrupts extracellular matrix interactions and causes dominant familial osteochondritis dissecans. Am J Hum Genet. 2010;86(2):126–37. 20137779 10.1016/j.ajhg.2009.12.018 PMC2820178

[20] Stattin EL, Lindblom K, Struglics A, Önnerfjord P, Goldblatt J, Dixit A, et al. Novel missense ACAN gene variants linked to familial osteochondritis dissecans cluster in the C-terminal globular domain of aggrecan. Sci Rep. 2022;12(1):5215. 35338222 10.1038/s41598-022-09211-y PMC8956744

